# Polish-German preschoolers develop and use heritage Polish differently depending on whether they heard German from birth or not

**DOI:** 10.3389/fpsyg.2023.1080122

**Published:** 2023-03-28

**Authors:** Annick De Houwer

**Affiliations:** Harmonious Bilingualism Network (HaBilNet), Rixensart, Belgium

**Keywords:** Polish, German, children, bilingual, language proficiency, language choice, preschool, parents

## Abstract

This study assessed the language proficiency and use of a hitherto under-investigated group, *viz*., 3.5-year-olds growing up with Polish as a heritage language and German as societal language. All children (*N* = 28) heard Polish from birth in the home but half the children also heard German from birth (Bilingual First Language Acquisition, BFLA) while the other half added German through preschool (Early Second Language Acquisition, ESLA). All children attended German preschools. Data collection relied on an online survey filled out by 28 mothers and 20 fathers. There were large discrepancies between parental answers to general versus detailed questions regarding language use (choice) amongst parents and children. This has important repercussions for much of questionnaire based bilingualism research. Children were developing productive language as expected but BFLA preschoolers spoke German better or spoke both languages equally well whereas ESLA preschoolers spoke Polish better. Apart from BFLA children’s much longer and daily exposure to German from birth, these BFLA-ESLA differences in relative Polish proficiency may relate to different current patterns of language choice, with (1) Polish less present in parent–child interactions involving BFLA than ESLA preschoolers, and with (2) BFLA but not ESLA preschoolers mostly hearing Polish from just a single parent. The BFLA-ESLA difference thus made a difference to children’s heritage Polish development and use already at age 3.5.

## 1. Introduction

This introduction sets the scene for the empirical study to follow. It reviews several studies of non-societal language use (henceforth: heritage language, HL^1^) by children under age 12 (section 1.1) and factors supporting or threatening that use (section 1.2). Most of the relevant studies concern children over age 4.5. Yet HL use prior to that age may already reveal some of the dynamics we find in older children. The current study therefore focuses on 3.5-year-olds. As discussed in section 1.3, one major factor supporting or threatening HL use consists of parental home language choice patterns since children were born, i.e., did parents speak both a HL and the societal language (SocL) at home, or solely the HL? Section 1.4 explains how the questionnaire study reported on in this article investigates this factor for a hitherto infrequently studied population, i.e., Polish-German preschoolers. Amongst others, the questionnaire included both general and detailed questions about patterns of home language choice. Section 1.5 explains the reasoning behind this. Section 1.6 lists the research questions.

### 1.1. Patterns of heritage language use in early and middle childhood

Portes and Hao (1998) report that “the majority” (p. 273) of their large adolescent sample in the USA could not speak their parents’ language. Large surveys reporting on bilingually reared younger children from across the world (Australia, Belgium, Canada, France, Japan) reveal massive intergenerational language loss of whatever HL they hear at home (De Houwer, 2020b): A fifth up to a quarter of bilingual school children may understand their HL but do not speak it. Smaller scale reports on primary school children (HLs-Arabic and Amazigh in Spain: Moustaoui, 2021; HL-Hebrew in the USA: Kaufman, 2001; HL-Japanese in the UK: Okita, 2002; HL-Russian in Germany: Anstatt, 2009; HL-Spanish in the USA: Anderson, 2012 and Buac et al., 2014) confirm these global findings. Furthermore, bilingually reared primary school children may speak their HL markedly less well than the SocL they hear at school (HL-Bangla in the UK: Al-Azami, 2014; HL-English in French-speaking Canada and Poland: Leśniewska and Pichette, 2018; HL-Japanese in the UK: Gyogi, 2015; for opposite findings, though, see HL-Russian in Israel and the Netherlands: Meir and Janssen, 2021, and HL-Russian in Cyprus, Ireland, Israel, and Sweden: Otwinowska et al., 2021). Bilingual primary school children may also show a different course of development for particular grammatical HL features than age-matched peers who speak that HL as their only language (HL-Hebrew in the USA: Kaufman, 2001; HLs-Polish and Portuguese in Germany: Rinke et al., 2019; HL-Portuguese in Germany: Flores et al., 2017; HL-Russian in Germany: Anstatt, 2009; HL-Russian in Israel and the Netherlands: Meir and Janssen, 2021; HL-Russian in Norway: Rodina and Westergaard, 2017).

Likewise, younger bilingual children may do less well in the HL than in the SocL. Twenty children between 4;5 (years;months) and 5;9 with HL-Polish in the UK did markedly less well on a Polish than an English lexical task (Abbot-Smith et al., 2018). Half of 89 mothers of sequential bilinguals aged 4;2 to 5;6 in Canada with SocL-English and a variety of different HLs reported “attrition in their child’s L1 abilities and a preference for English compared to the L1” (Sorenson Duncan and Paradis, 2020, p. 52). A three-year longitudinal study of HL lexical and grammatical development in 34 HL-Spanish bilingual children in the USA who were on average aged 4;2 at the beginning of the study showed many patterns, including HL growth as well as HL attrition and loss, with some children hardly being able to speak the HL by age seven, although they spoke the SocL fluently (Hiebert and Rojas, 2021). The fact that HL performance can decline with age was also shown by Armon-Lotem et al. (2021), who found that older (ages 6;0–6;5) HL-English children in Israel scored worse on monolingual-based English tests than younger (ages 5;0–5;5 and 5;6–5;11) peers. Except for narrative skills, 88 bilinguals aged 4 to 7 (mean: 5;8) with HL-Polish in the UK had much lower Polish scores on several tests compared to monolinguals peers in Poland (Haman et al., 2017). The gaps remained the same regardless of age. Mieszkowska et al. (2017) found that 14 HL-Polish bilinguals and 14 HL-Polish trilinguals between ages 4;5 to 6;7 (mean: 5;6) in the UK did worse on standardized picture-naming and word-recognition tests compared to 14 age matched Polish monolinguals in Poland.

Like children in middle childhood, preschoolers may show a different course of development for particular HL features than age-matched peers who speak that HL as their only language. Schwartz et al. (2015) demonstrated this for 70 HL-Russian sequential bilinguals in Israel aged 4 to 5: Noun-adjective gender agreement error patterns were qualitatively similar for bilinguals and monolinguals, but quantitatively bilinguals resembled younger monolinguals rather than age-matched peers. Klassert et al. (2014) showed similar effects for HL-Russian noun naming by 60 Russian-German sequential bilinguals aged 4–7. Also in Germany, Brehmer and Rothweiler (2012) showed that German-Polish bilinguals had not completely acquired HL-Polish attributive adjective gender assignment by age 6.5, an unexpected result compared to Polish monolingual children.

On the other hand, preschoolers with exposure to both a HL and a SocL from birth often show similar morphosyntactic development compared to monolinguals peers in either language, although also within this population uneven development across languages is quite common. Children exposed to two languages from birth are growing up in a Bilingual First Language Acquisition or BFLA setting (Meisel, 1989; De Houwer, 2009, 2021).

HL vocabulary size has been at focus in a handful of studies on toddlers. Fifty-three bilingual toddlers in the UK and Ireland with HL-Polish had smaller Polish expressive vocabulary sizes than age matched monolingual peers in Poland (Miękisz et al., 2017). On the other hand, 31 toddlers aged 1;1 and 1;8 with HL-French in Dutch-speaking Flanders performed well within monolingual norms or even better (De Houwer, 2010). Rinker et al. (2017) found greater HL-Turkish than SocL-German production vocabulary for 19 children in Germany between 2;0 and 3;6 (most were BFLA). Ninety-two younger bilinguals (aged 1;6 to 2;6) in Germany produced up to three times as many HL-Turkish as SocL-German words (Budde-Spengler et al., 2021).

The above overview reveals that studies mostly concern primary school children or older preschoolers (starting in the fifth year of life). So far, few HL studies have concentrated on young preschoolers, that is, children aged three to four. It remains to be seen to what extent HL use in that younger population already shows signs of attrition or slower development.

### 1.2. Some explanations for patterns of HL use in early and middle childhood

Studies have investigated various factors to help explain patterns of HL use. Parents in the UK rated 18 HL-Polish 5.5-year-old bilinguals as having lower HL-Polish skills than parents of 18 peers in Norway (Hansen et al., 2019). There is some evidence that HL development patterns may be related to the specific languages involved. Czapka et al. (2021) undertook a longitudinal study of HL lexical development in 147 HL-Turkish or HL-Russian bilinguals in Germany who at pretest were on average aged 3;3; 119 children were still in the study by the fourth and last study wave, when children were on average aged 5;6. Results from a picture naming task showed different lexical growth trajectories for HL-Turkish and HL-Russian: At the last test point, lexical abilities were lower in the former. On the other hand, Rinke et al. (2019) found no differences between HL-Portuguese and HL-Polish direct object realization in 8-year-old bilinguals in Germany. Conversely, HL development patterns may be related to which SocL children are acquiring: Schwartz et al. (2015) found fewer HL-Russian gender agreement errors in sequentially bilingual preschoolers who were additionally acquiring a language with gender agreement than those who were not (but see Rodina et al., 2020, for a comparative five country study that failed to find an effect of the local SocL on older bilingual children’s HL-Russian gender assignment).

Aside from the specifics of the particular languages involved, HL development may be affected by the age at which children started acquiring the SocL, with a later age supporting the HL (Armon-Lotem and Ohana, 2017; Armon-Lotem et al., 2021; Czapka et al., 2021; Meir and Janssen, 2021). Within a group of 457 children in Singapore aged between 4;1 and 6;6 with either Malay, Mandarin, or Tamil as HL those with lower proportions of HL home exposure had lower HL vocabulary sizes (Sun et al., 2020). In the realm of sentence interpretation, 32 children aged 6 to 12 with HL-Greek in the USA who had used the HL more before age five as well as concurrently did better than peers who had done so less (Chondrogianni and Schwartz, 2020). Sun et al. (2022) reported similar findings for 202 4- to 5-year-olds with HL-Mandarin in Singapore. In a very large population study in Spain, Caminal et al. (2021) showed that increased parental HL-Catalan proficiency led to parents speaking the HL more often with their firstborn children. Likewise, but now for 294 4- to 5-year-old bilinguals with HL-Mandarin in Singapore, Sun et al. (2022) found that higher maternal HL proficiency was associated with more frequent HL talk to their children. Rinker et al. (2017) found the same for HL-Turkish-speaking parents of two- and three-year-olds in Germany. Importantly, Sun et al. (2022) also found that maternal HL proficiency was related to children’ receptive HL skills: The more HL proficient mothers were, the better children’s HL performance. Mieszkowska et al. (2017) attributed greater SocL-English than HL-Polish vocabulary size in 14 bilinguals and 14 trilinguals between ages 4;5–6;7 to differences in the relative exposure to either language, with the SocL being more strongly present in children’s lives. Haman et al. (2017) found that cumulative HL-Polish exposure helped explain children’s production but not their comprehension skills. Budde-Spengler et al. (2021) found that higher parental education was related to toddlers’ greater HL-Turkish vocabulary size.

Studies cited so far have mostly relied on parental reports as regards children’s HL exposure. Studies focusing on observational data include Gaskins (2020), which found that high numbers of early verbs in two BFLA toddlers’ HL-Polish in the UK could be traced to children hearing inflected Polish verbs in isolation and at the beginning and end of utterances more frequently than their uninflected English counterparts. Gaskins and Frick (2022) suggested that early multimodal interactions with two HL speakers may facilitate early HL development. De Houwer and Nakamura (2022) reviewed how parental responses to children’s language choice can encourage children’s HL use. However, children may resist such parental socialization patterns through discourse and insist on speaking solely the SocL (for potential explanations of such resistance, see De Houwer, 2020b). Sibling SocL use at home may lead to less HL talk by younger siblings (Mirvahedi and Cavallaro, 2020). As discussed next, a major explanation for patterns of HL development in children may relate to long-term parental language choice patterns.

### 1.3. A focus on parental language choice/use patterns and associated acquisition setting: BFLA and ESLA

Rodina and Westergaard (2017) found an influence of what they called “family type” in terms of parental home language use, here termed “language choice.” They distinguished between families with children aged between 4;3 and 7;11 where both parents spoke just HL-Russian (*N* = 10) and so-called “mixed” families, where parents spoke both HL-Russian and SocL-Norwegian (*N* = 10) in Norway. Children’s HL development was more advanced when both parents spoke just the HL at home. Rodina et al.’s (2020) study of 209 bilingual HL-Russian children between ages 3;0 and 10;0 (mean age around 6;0) in five countries confirmed this finding (this author calculated that 46% of children grew up in bilingual homes, that is, homes where parents spoke both a HL and a SocL). One can surmise that children who heard both languages from their parents at home had done so from birth, and were thus growing up in a BFLA setting. Parental language choice patterns in BFLA families are usually established when children are born, and do not change much in children’s preschool years (De Houwer and Bornstein, 2016).

The 10 children in Rodina and Westergaard (2017) and the 55 children in Norway in Rodina et al. (2020) who heard just the HL at home started being exposed to the SocL at age one. The 154/209 children outside Norway in Rodina et al. (2020) started being exposed to the SocL at age three. All these children were experiencing an Early Second Language Acquisition (ESLA) setting (De Houwer, 1990, 2021), where regular exposure to a second language takes place after a period in which children under age six were acquiring just a single language in a monolingual family. Typically, such exposure arises through attending group child care or preschool in the local SocL. We know little about any changes over time in parental language choice patterns in ESLA families (De Houwer, 2021) but anecdotal reports mention that parents may add the SocL in their interactions with children in children’s school years (De Houwer, 2020a).

The fact that Rodina and Westergaard (2017) and Rodina et al. (2020) found an influence on children’s HL of “family type” is in line with findings based on a large (*N* = 1,778) investigation in Dutch-speaking Flanders of the influence of parental home language choice on child HL development (De Houwer, 2007; all families here had at least one child aged 6 to 9 who was attending school in the SocL). The five logically possible patterns of parental home language choice were all present: (i) parents both spoke just the HL, (ii) both parents spoke the HL but one parent in addition spoke the SocL, (iii) both parents spoke both the HL and the SocL, (iv) one parent spoke the HL and the other one the SocL, and (v) both parents spoke the SocL and one parent the HL^2^. Rodina and Westergaard’s (2017) and Rodina et al.’s (2020) “only the HL at home” coincides with pattern (i); their “mixed” category covers patterns (ii) through (v). Children growing up with pattern (i) are growing up in a monolingual family and acquiring the SocL as a chronologically second language, either through child care or preschool (ESLA), or through school after age six, in a Second Language Acquisition setting, when children start learning a new language in the spoken but also in the written mode (De Houwer, 2021). Children growing up with patterns (ii) through (v) are growing up within a bilingual family, presumably from birth, so in a (likely) BFLA setting^3^.

De Houwer’s (2018b) re-analysis of her 2007 survey data showed that only 70% of BFLA children spoke their HL. In contrast, (E)SLA children, who exclusively heard the HL at home, spoke the HL in 97% of (E)SLA families^4^. This large BFLA/(E)SLA difference points to the importance of parental language choice patterns in the home for explaining children’s HL use in the primary school years, with the (E)SLA setting better supporting HL use, and confirms that “family type” is an important category for helping to explain HL development.

Whether the crucial point is that in monolingual families both parents speak the HL, as Rodina and Westergaard (2017) and Rodina et al. (2020) suggest, is another matter. De Houwer (2007) found no difference between “mixed” families where both parents spoke the HL and additionally one parent spoke the SocL (pattern ii), on the one hand, and families where both parents spoke just the HL (pattern i), on the other, in terms of whether they had children who spoke the HL or not. Furthermore, “mixed” families where both parents spoke both the HL and the SocL (pattern iii) had just as low a chance of having children who actually spoke the HL as “mixed” families where only one parent spoke the HL and the other one the SocL (pattern iv). (Pattern (v) families had the lowest chances of having a child who spoke the HL.)

The 70–97% difference between BFLA families on the one hand and (E)SLA families on the other (De Houwer, 2018b) suggests that it is the BFLA-ESLA difference rather than whether two parents speak the HL at home that is of fundamental importance. Although studies of young children’s HL development may collect data on both BFLA and ESLA children and investigate the role of exposure (e.g., Armon-Lotem and Ohana, 2017; Haman et al., 2017; Hansen et al., 2019) virtually none examine the extent to which exposure to the HL and the SocL from birth has a potentially different effect than if exposure to the SocL happened only after children were in a monolingual home environment for some time. The main goal of the present study is to examine the influence of a BFLA versus an ESLA acquisition setting on bilingual children’s HL development.

We know that the difference between BFLA and ESLA plays a major role for the HL once children are in primary school (see above). Budde-Spengler et al. (2021) found no differences between HL-Turkish production vocabulary size in toddlers up to age 2.5 in Germany who heard both HL-Turkish and SocL-German (BFLA) or only HL-Turkish (ESLA) at home. It is possible that age 2.5 is too young for BFLA-ESLA differences to show up. The current study with children who were a year older (around age 3.5) examines the extent to which an influence can be seen at early preschool age, thus keeping chronological age and overall time for acquiring the HL constant. A focus on young preschoolers fills a gap in the research literature on HL development, where, as reviewed above, it is virtually absent^5^.

Within the broad distinction between BFLA and ESLA families there might be additional family language choice patterns that are of importance. Parental home language choice patterns may have changed in the course of the early years. This may have happened in response to children’s own language choice patterns. For instance, once children start attending child care or preschool in the SocL they may add the SocL in interaction with parents with whom they were previously solely speaking a HL (De Houwer, 2017a). This in turn might lead parents to adjust their own language choice patterns, away from the HL (De Houwer, 2020a). Another reason for changes in home language choice patterns may be families’ increased experience with living in a language contact situation, with the associated need to adjust to people outside the family. HL-speaking parents may also become more proficient in the SocL, increasing the chance they will start speaking (more of) the SocL at home. Before any reasons for possible changes in family language choice patterns can be investigated, however, one needs to know what these patterns consist of. This is why this study also considers family language choice patterns.

### 1.4. Substantive contribution: A focus on HL-Polish with German as SocL

In this likely first systematic comparison of BFLA and ESLA preschoolers’ HL use and experience, the focus is on Polish-German-speaking children and their families with HL-Polish and SocL-German^6^. Data were collected in Germany and Austria, where German is the SocL used in public life, education institutions, and in child care and preschool. This study is part of a larger project on early bilingualism involving SocL-German that examines HL-English as well, in a bid to investigate the potential of HL status differences on HL development. English is a high status language in German-speaking countries, whereas Polish is not (Plewnia and Rothe, 2011). The decision to focus on HL-Polish rather than other low status HL-languages in Germany such as HL-Russian or HL-Turkish (Plewnia and Rothe, 2011) was founded in the existence of several other studies on those HLs in Germany (see review above) and on the scarcity of studies there focusing on early HL-Polish development (see below). Thus this study makes a substantive contribution as well, uncovering realities of language use and development within bilingual Polish-German preschoolers.

In Germany people of Polish descent represent the second largest group of people with a migration background (Statistisches Bundesamt, 2020). In Austria individuals of Polish descent represent the seventh largest immigrant group (Statistik Austria, 2020). The brief literature overview here focuses on HL-Polish in Germany only. Relevant sources for Austria were not found.

Polish is widely spoken in Germany (Brehmer and Mehlhorn, 2020; Brehmer and Sopata, 2021). HL-Polish has been studied in primary school aged children (Rinke et al., 2019), adolescents (Brehmer et al., 2016; Brehmer, 2017), older teens (Anstatt, 2013; Besters-Dilger et al., 2015; Pułaczewska, 2019), and adults (Brehmer and Czachór, 2012; Besters-Dilger et al., 2015; Anstatt and Mikić, 2022).

Studies involving Polish-German bilingual children under age six living in Germany are few and far between (Table 1, order of studies according to date of publication). Studies focused on the HL or the SocL (or both). Group studies in Table 1 are difficult to interpret because they collapsed data for several HLs and/or for younger and older children, possibly masking important age related differences. They combined BFLA and ESLA children within their analyses or did not indicate whether children acquired both languages from birth or not. This makes it impossible to assess the effect of a BFLA vs. an ESLA setting on HL-Polish development. Where relevant, findings from studies in Table 1 are cited in the Discussion section to the current study.

**Table 1:** Studies involving Polish-German preschoolers in Germany *.

Study	*N* children	Age(s)	Focus	Comments	Information re BFLA/ESLA?
Reich (2009)	6	Anywhere between 3;6–6;9	Overall HL and SocL development	Part of group of 36 children with additional HLs; no separate analyses for Polish	No
Sopata (2011)	4	Anywhere between 2;8–5;8	Use of SocL infinitives	10-month long longitudinal observations per child	ESLA
Schneider (2012a)	2	(1) 1;0–9;0 (2) 4;0–12;0	Language choice patterns between children, amongst family members, and outside the home	8-year-long in depth double case study of two brothers (extension of Schneider, 2012b)	BFLA
Schneider (2012b)	2	(1) 1;0–5;0 (2) 4;0–8;0	Interactions between children	4-year-long double case study of two brothers (zooming in on part of the data discussed in Schneider, 2012a)	BFLA
Brehmer and Rothweiler (2012)	34	2;11–6;5	HL gender marking on attributive adjectives (*cf*. section 1.1)	Two elicitation tasks	Yes but not taken into account in analyses
Kulik (2016)	13	“Zwischen dem fünften und sechsten Lebensjahr” (p. 105) = between the 5th and 6th year of life	HL-Polish morphosyntactic proficiency (the use of case, verb morphology, and coordinate vs. subordinate clauses) and cross-linguistic transfer	Based on children’s picture descriptions; part of a larger study with older children; also some overall parental assessments of children’s proficiency in each language; no developmentally oriented analyses	No but all families used both the HL and the SocL (to different extents)
Schaefer et al. (2019)	15	Between 3;5–4;10	Comprehension of 20 nouns and verbs in HL-Polish, HL-Turkist, and SocL-German	Results analyzed in combination with data from 21 Turkish-German peers	No
Sopata and Putowska (2020)	29	Between 4 and 11, mean 7;3	Children’s language choice patterns and overall percentage of “correctness” of children’s HL-Polish in an elicitation task	No separate analyses for preschoolers	No
Sopata and Długosz (2022)	58	Between 4;11 and 13;9, mean 9;3	Grammatically correct performance on a SocL sentence repetition task	No separate analyses for preschoolers	No
Jachimek et al. (2022)	1	1;4–4;0	Longitudinal study of the use of modifiers in HL-Polish and SocL-German noun phrases		BFLA

* Participant age ranges in Sopata et al. (2021) and in Brehmer and Sopata (2021) suggest that these studies included at least one or two Polish-German preschooler(s) but lack further information.

### 1.5. Methodological contribution: A focus on different ways of asking the same thing

Most of the studies reviewed above rely on responses to parental questionnaires. They take parental responses about home patterns of language choice at face value. However, the author’s experience in working with many bilingual families over several decades has often laid bare discrepancies between parental claims about their and their children’s language choice patterns on the one hand, and actual practices on the other. This is why in the design of the study both general and detailed questions about language choice were included. Detailed questions focus the participants’ attention more, and give pause to reflect better. Thus some discrepancies in comparison with more general questions are to be expected.

Studies relying on parental responses to questionnaires usually do not state which parent(s) filled out the questionnaire. The present study aimed particularly to involve both parents, thus adding a level of reliability. The addition of information supplied by both parents may, however, lead to additional discrepancies with information provided by a single source.

### 1.6. Research questions

To summarize, this study aims to answer the following research questions:

(RQ1) does growing up in a BFLA vs. an ESLA family make a difference for HL development and use in 3.5-year-olds?
(RQ2) what are the patterns of HL-Polish development and use amongst 3.5-year-olds and their parents in a German-speaking society, and do these patterns differ depending on family type (BFLA or ESLA)?
(RQ3) are parental answers to general questions about language choice mostly in line with answers to detailed questions about language choice?

## 2. Method

### 2.1. Instruments

This study is part of a longitudinal study on Polish-German and English-German early bilingualism. Data were collected around children’s second birthdays (Wave1), 9 months later (Wave2), and another 9 months later (Wave3).

The present study concerns Wave3 data for families who spoke Polish at home collected through the Polish-German and English-German Early Bilingualism Online Survey-3 (PEGEBOS-3; De Houwer, 2017b). PEGEBOS-3 centered on parents’ 3.5-year-old children, family composition, residences, vacations, child care arrangements, and overall patterns of family language use. The analyses below discuss responses to parts of PEGEBOS-3 and to selected components of BILTALK, Talk and Interaction Questions for Parents in Bilingual Settings (De Houwer, 2018a), a sub-questionnaire asking both mothers and fathers on a more detailed level about language interaction with 3.5-year-olds. BILTALK also queried children’s language proficiency. There were also questions about using both languages in a conversation and sentence (not covered here). Questionnaire items are further described below where relevant.

PEGEBOS-3 exists in English and Polish. BILTALK additionally exists in German. The present study relies on German and Polish versions. When in the following survey items are mentioned in English, they represent translations of German or Polish equivalents.

### 2.2. Respondents

The focus is on 28 families within the larger study who contributed data at each of the three Waves. Respondents were parents of children who had been regularly hearing Polish and German from birth within the family (BFLA families, *N* = 14) or Polish only (ESLA families, *N* = 14), as declared at recruitment.

Families were recruited after parents took the initiative to contact the research team in response to announcements through playgroups, Facebook groups, and word of mouth that we were looking for families to participate in a study on early Polish-German bilingualism. Team members of Polish descent who were mothers of Polish-German toddlers had an extensive recruitment conversation with potential participants. Families who agreed to participate signed an informed consent form. It took nearly 2.5 years to recruit families who fulfilled the conditions and who were willing to invest their time and effort in the study over 1.5 years.

At recruitment target children were nearly 2 years old. For the ESLA families it was crucial that children had (or would have) the opportunity to regularly hear German outside the home. Parents were only recruited as an ESLA family if their toddler was attending a German-speaking preschool or parents confirmed they were planning on soon sending their child to such a preschool^7^.

Families lived scattered throughout Germany (25) or Austria (3) at recruitment and for the duration of the study. In 13 BFLA families mothers had emigrated from Poland; in one BFLA family it was the father who had done so. Their partners were German speakers who had always lived in Germany or Austria. The larger presence of mothers of Polish descent in the mixed origin families reflects the fact that in Germany many more Polish women marry German men than the other way around (Pułaczewska, 2019). All BFLA target children were born in Germany (12) or Austria (2). All ESLA parents had emigrated from Poland. Nine ESLA target children were born in Germany; the remaining five were born in Poland but moved to Germany before age one.

At recruitment, most families consisted of mother, father, and at least one toddler (the target child); one ESLA mother was raising her toddler alone. There was no difference between BFLA and ESLA mothers’, *t*(26) = 0.720, *p* = 0.478, or fathers’ ages, *t*(26) = 0.270, *p* = 0.789 (Table 2, A).

**Table 2:** Family demographic information (sections 2.2 and 2.3).

	BFLA families	ESLA families
Total *N*	14	14
Parents who emigrated to Germany or Austria from Poland	14	27
Target children lived with both mother and father	14	13
**A**. Parental average age at recruitment		
Mothers	33.93	35.21
Fathers	38.69	37.14
**B**. Parental education (highest degree)		
Parents with a doctoral degree	7	2
Parents completed a Master’s program	18	17
Parents completed a four-year college program	1	1
Parents completed secondary school	1	7
Parents completed middle school	1	0
**C**. Parental work status		
Fathers worked full-time outside the home	12	13
Mothers worked full-time outside the home	3	1
Fathers worked part-time outside the home	2	0
Mothers worked part-time outside the home	5	5
Mothers did not work outside the home	6	7
Maternal work status unknown	0	1
**D**. Parental reported language proficiency		
Mothers…	14	14
…fluently spoke both Polish and German	13	5
…fluently spoke Polish and could manage in German	0	4
…fluently spoke Polish but hardly spoke any German	0	5
missing information	1	0
Fathers…	14	13
…fluently spoke both Polish and German	4	7
…fluently spoke Polish and could manage in German	0	3
…fluently spoke Polish but hardly spoke any German	0	3
…fluently spoke German and could manage in Polish	4	0
…fluently spoke German but hardly spoke any Polish	5	0
missing information	1	0
Mothers and fathers combined…	28	27
…fluently spoke both Polish and German	17	12
…fluently spoke Polish; spoke German (much) less well	0	15
…fluently spoke German; spoke Polish (much) less well	9	0
missing information	2	0
**E**. Children started attending preschool…		
Before age 3	11	8
Around age 3	3	4
Missing age data	0	2
**F**. Target children’s sibling status at Wave3		
Only child	4	1
Firstborn	5	4
Older sibling(s)	3	7
Older and younger sibling(s)	2	2
**G**. Any family trips longer than a week between children’s third birthday and Wave3?		
No	5	4
Yes, but no trips to Poland	2	1
Yes, including a single trip to Poland	5	5
Yes, including two trips to Poland	1	2
Yes, including three trips to Poland	1	2

Generally, parents were highly educated (Table 2, B). Considering the difference between seven BFLA parents with a doctoral degree and seven ESLA parents with just secondary school BFLA parents were more highly educated than ESLA parents. All children except two in an ESLA family were growing up with at least one parent who had completed at least a Master’s program.

At Wave3 most fathers were working full-time outside the home (Table 2, C). Mothers’ employment was more variable. Parental employment status was similar for BFLA and ESLA families but differed mostly for fathers and mothers, with 26 of the former but only four of the latter working full-time outside the home.

Information supplied at Wave2 (Table 2, D) showed that 13 BFLA but only five ESLA mothers were fluent speakers of both Polish and German; this includes the German mother living with a Polish origin father. All BFLA fathers were fluent in German (including the Polish origin father) and had variable Polish proficiency. All ESLA parents spoke Polish fluently and had variable German proficiency. In one ESLA family no parent could speak German. More ESLA than BFLA parents fluently spoke Polish and more BFLA than ESLA parents fluently spoke German, *χ*^2^(1, *N* = 82) = 7.253, *p* = 0.007.

### 2.3. Target children and family experience

Most families (12 ESLA, 10 BFLA) filled in the survey when target children were very close to age 3.5 (range: 3;5.20 [years;months.days] – 3;6.29). Five families (3 BFLA, 2 ESLA) did so a bit later (range: 3;6.30–3;8.23), and one BFLA family several months later (age 3;11.23). Although not all children were strictly speaking 3.5 years old (4 BFLA and 2 ESLA children were a bit older), they will be referred to as such.

All children were attending preschool by Wave3 (Table 2, E), most for about 30 h a week. Children occasionally heard staff speak additional languages than German, but these did not include Polish. All children were singletons (15 girls, 13 boys). There was no BFLA-ESLA difference in children’s gender distribution, *χ*^2^(1, *N* = 28) = 1.292, *p* = 0.256. PEGEBOS-3 asked whether there had been any serious health issues since children’s third birthdays. None except one were reported (the exception was an ESLA child who underwent a hernia operation 4 months before Wave3).

In nine BFLA families, the first child they were raising bilingually was their 3.5-year-old (Table 2, F). In contrast, in nine ESLA families their 3.5-year-old was likely not the first child parents were raising with Polish in a German-speaking environment (PEGEBOS-3 did not query where older siblings grew up; they might have lived in Poland before this study’s target children were born). In any case, as a group, BFLA and ESLA parents had had different experiences with (bilingual) parenting:

PEBEGOS-3 asked about any trips longer than a week that families took since their 3.5-year-olds’ third birthdays (Table 2, G). No BFLA-ESLA differences in family travel to Poland emerged, *χ*^2^(1, *N* = 27) = 0.898, *p* = 0.445.

### 2.4. Procedure and respondent coding

Families lived throughout a very large geographical area, most at great distance from the research team’s base in central Germany. This rendered it impossible for families to come to the university for language tests or for researchers to make individual home visits, the latter also being logistically impossible because of the study’s long length of time (full data collection for the entire sample took 4.5 years). Resources thus dictated the decision to run the study online (and through paper correspondence for aspects not reported here).

Parents were invited to fill in the online survey through an individualized email link, with the request to complete it within 2 weeks. Participants who were late were sent a reminder. The fact that some children were a bit older than 3.5 relates to some parents taking their time in completing the survey, in spite of several friendly reminders. Parents were free to choose in which language they wanted to fill in the survey (Polish or German).

It took about 20 min to complete PEGEBOS-3 and an additional 15 min to complete BILTALK. After completion of the survey through the online platform SurveyMonkey (as well as the return of additional instruments on paper used in this study, not reported here), target children were sent a small age appropriate gift together with a thank you letter for the parents. Parents greatly appreciated the gifts, as communicated to the relevant research assistants. Once data had been collected from all the participants in the study a lottery took place in the research team’s office, after which six randomly drawn families received an additional children’s gift. All families received pictures of the lottery “happening” and a final thank you note.

Parent reporters were identified as Polish- or German-speaking. This was done on the basis of the language parents indicated they generally addressed to their 3.5-year-old, regardless of parental proficiency in the other language or detailed home language choice patterns as evidenced by responses to the BILTALK sub-questionnaire (section 3.2). In all BFLA families except one each parent spoke either Polish or German with 3.5-year-olds. One BFLA mother regularly spoke both languages with her son. She was identified as Polish-speaking because she was the only source of Polish input to her son within the family. In all ESLA families except one each parent spoke Polish with 3.5-year-olds. In the exceptional ESLA family both parents addressed their only child in both languages. Yet they had spoken only Polish to their child earlier. This is why they were both identified as Polish-speaking.

All BFLA Polish-speakers (13 mothers and one father) and all ESLA Polish-speakers (14 mothers) filled out the entire PEGEBOS-3 survey, including BILTALK. They did so in Polish. Nine ESLA Polish-speaking fathers and 11 BFLA German-speaking parents (10 fathers and one mother) completed only the BILTALK sub-questionnaire (Table 3). The Polish-speakers did so in Polish; the German-speakers in German. For BILTALK a total of 28 mothers and 20 fathers (11 BFLA, 9 ESLA) supplied data. Mothers filled it out first. Parents were asked to fill out BILTALK without consulting the other parent.

**Table 3:** Who filled out the BILTALK sub-questionnaire?

	BFLA	ESLA	Total
2 Polish-speakers	n.a.	9	9
1 Polish-speaker and 1 German-speaker	11	n.a.	11
1 Polish-speaker only	3	5	8
Number of children reported on	14	14	28
Minimum number of expected responses per BILTALK survey item (=number of parents who supplied data)	25	23	48

n.a., not applicable.

## 3. Analyses and results

Following the research questions (section 1.6), analyses were geared towards investigating differences and similarities between BFLA and ESLA children and their families on various measures. They started with an examination of children’s reported language proficiency. Afterwards, the focus was on family language choice patterns.

### 3.1. Children’s language proficiency

Six BILTALK items concerned children’s language proficiency (see Appendix A for the items, response categories and ordering). Two queried language comprehension (one item per language). Four concerned production. Like in the parental questionnaire developed by Gagarina et al. (2010), parents were asked to evaluate their children’s comprehension and production skills in each language on a Likert scale (see also Meir and Janssen, 2021).

#### 3.1.1. Comprehension

Parents were asked to what extent they agreed with items PR1 and PR3 in Appendix A (*cf*. When I talk to my child in Polish/German, (s)he often expresses misunderstanding of a word or phrase). As shown in Table 4, most parents who felt they could judge it entirely disagreed with these statements (29/38 responses for HL-Polish; 19/23 responses for SocL-German). Four BFLA and six ESLA children received the less favorable ratings *Not quite agree* or *More or less agree* by at least one parent for at least one language. On the whole, then, comprehension issues were few and far between. There were no differences in parental responses between languages, *χ*^2^(1, *N* = 61) = 0.338, *p* = 0.561, or between BFLA and ESLA parents, *χ*^2^(1, *N* = 61) = 1.009, *p* = 0.315.

**Table 4:** Comprehension misunderstandings: Number of parental responses per language.

	BFLA	ESLA
**A.** HL-Polish		
Completely agree	0	1*
More or less agree	1	0
Not quite agree	3	5
Entirely disagree	12	17
Does not apply, I do not speak any Polish to my child	9	0
**B.** SocL-German		
Completely agree	0	0
More or less agree	0	1
Not quite agree	1	2
Entirely disagree	14	5
Does not apply, I do not speak any German to my child	10	11
No response	0	4

* This response by a Polish-speaking father must be in error, given that all other responses by this father point to quite fluent usage of HL-Polish by his child. It was treated as “No response.”

Focusing just on HL-Polish, one ESLA child received ratings by both parents that might be indicative of (light) problems with HL comprehension: Both parents gave a *Not quite agree* rating. Two additional ESLA children and one BFLA child received only maternal ratings on the comprehension statements (*Not quite agree*). For three other children (two BFLA, one ESLA) maternal *Not quite agree* ratings were set off by paternal *Entirely disagree* ratings. One BFLA girl received a rather bad maternal *More or less agree* rating (given that she was rated by her father as not having any comprehension issues in German, she is not to be seen as a “problem case” for comprehension development as a whole).

#### 3.1.2. Production

##### 3.1.2.1. Structural complexity

Two items (PR4 and PR6 in Appendix A) queried the complexity of language production, one for each language. While 3.5-year-olds are still fully in the process of acquiring language, they can normally already produce complex sentences and thus potentially show developmental differences between languages that will be clear to parents. Parents rated how often they had heard children say fairly complex sentences like three sample utterances in each language (Table 5). Parents used all five answer categories available to them (Appendix A), thus allowing distinctions between ratings (and hence children).

**Table 5:** Sample sentences in language complexity questions in BILTALK.

Polish sample sentences	*English glosses*	German sample sentences	*English glosses*
a jak wiewiórka umrze, to gdzie idzie?	*and if the squirrel dies, where does it go?*	erst muss ich mich mal richtig hinlegen	*first I have to lie down properly*
ja najpierw muszę przyjść do pani	*I have to come to you first*	komm, wir wollen dies gerade spielen	*come on, let us play this right now*
daj mi te pieniążki, bo ja będę zapłacać	*give me the money, because I will pay you*	Mutti, ich hätte im Spiel Fieber, weil meine Stirne sind ganz heiss	*mommy, I would have a fever in the game because my foreheads are very hot*

Parents were instructed not to pay attention to specific words and contents in the sample utterances, but to sentence structure. Utterances resembled those typically produced by Polish and German monolingual 3.5-year-olds, including some of their typical errors (see, respectively: Smoczyńska, 1985; Mills, 1985). Schneider (2012a, p. 63) reported similarly structured Polish and German utterances as said by her BFLA son in the fourth year of life.

A small pilot presented the sample utterances to Polish and German mothers in bilingual and monolingual families with 3.5-year-olds. Parents found them representative of 3.5-year-old speech. One would expect children to use them *frequently* or at least *regularly*. Children who *sometimes*, *hardly*, or *never* used them were comparatively not as highly developed. In a bilingual setting, well developed use in at least one language is sufficient to dismiss the possibility of an overall language learning delay (e.g., De Houwer, 2018b).

A first focus was on the extent to which children were rated as using complex structures in any language. The separate answers for each language were thus compared and the best score in any language tallied (Table 6, A).

**Table 6:** Language complexity ratings.

	BFLA	ESLA
**A**. Overall complexity: Best score by any parent in either language per child *		
Frequently	9	9
Regularly	4	3
Sometimes	1	1
Hardly	0	1
**B**. HL complexity: Ratings by Polish-speakers (27 mothers, one father) for 14 BFLA and 14 ESLA children		
Frequently	5	8
Regularly	5	4
Sometimes	2	1
Hardly	2	1
**C**. Comparison of parental complexity ratings (possible for 23 BFLA and 10 ESLA parents)		
Same complexity rating in both languages	12	3
Higher complexity rating for HL-Polish	4	6
Higher complexity rating for SocL-German	7	1
**D**. Cross-linguistic comparison of parental complexity ratings per child **		
Same complexity rating in both languages	5	1
Higher complexity rating for HL-Polish	3	5
Higher complexity rating for SocL-German	6	1

* Two BFLA German-speaking fathers did not answer the question about Polish complexity (one father did not speak Polish, the other one hardly so); most (13/23) ESLA parents stated they had not heard children use any complex German sentences. Ten of these parents were hardly fluent in German.

** Cross-linguistic comparative complexity ratings were available for all 14 BFLA children. Comparative complexity ratings were available for only 7 ESLA children because for the others no parent had supplied a German rating.

Most (13/14) BFLA children were rated by at least one parent as having an expected speaking ability (answers *frequently* and *regularly*) in at least one language. One girl’s best score in either language was that she only *sometimes* used the level of complexity in the sample utterances. Unfortunately data from the girl’s German-speaking father for a second opinion were lacking. All except perhaps one of the BFLA children, then, were *frequently* or *regularly* producing the kinds of complex structures expected for 3.5-year-olds in least one language.

The distribution amongst the best scores by any parent in either language for the ESLA children is virtually identical to those for the BFLA children (Table 6, A). For one ESLA girl both parents agreed she only *sometimes* used complex Polish structures. Parents had not heard her use any complex German structures (as later emerged from the language choice data, their daughter did speak some German with them, though). The mother who was single stated she had *hardly* ever heard her ESLA daughter use Polish complex structures and had *never* heard her child use complex German structures (her daughter only spoke Polish with her). Information from persons more familiar with these two ESLA children’s use of German would be needed to assess their overall level of language development. Much like the BFLA children, then, all except perhaps two ESLA children were *frequently* or *regularly* producing the kinds of complex structures expected for 3.5-year-olds in at least one language.

Summarizing, in at least one language, most bilingual preschoolers were able to use complex structures as expected for their age. For three of the less well performing children (1 BFLA, 2 ESLA) additional SocL-German ratings would be required to assess whether they were perhaps slightly delayed in their overall language development. BFLA-ESLA differences did not emerge.

Having established that at least 25 out of 28 bilinguals were producing complex sentences at a level expected for their age, the analysis now turns to complexity in the HL. For this it considers responses by Polish-speaking mothers only (Table 6, B). This is because mothers worked outside the home far less than fathers (section 2.2) and thus had more opportunity to hear children talk. Paternal rather than maternal ratings for the BFLA family where the father was the Polish-speaker were also included. No BFLA-ESLA differences emerged, *χ*^2^(1, *N* = 28) = 0.849, *p* = 0.357.

There was little overall variation amongst children in HL-Polish complexity ratings (Table 6, B). All the more noticeable were the five children (BFLA: 3, ESLA; 2) who did not receive a favorable rating (*sometimes* or *hardly* complex HL structures) from any Polish speaker (not just mothers). In a detailed visual inspection of the raw data, several factors were explored as possible explanations: whether children had older siblings (and perhaps heard more German from them than Polish), whether they had younger siblings (Polish-speakers with infants may have had less time to speak with 3.5-year-olds, offering children less HL input), or whether mothers worked outside the home. For the five more poorly performing children there was a great deal of variation in all these factors, rendering it impossible to discern any patterns.

Finally, for all children it was investigated whether family trips to Poland were associated with HL complexity (complexity ratings *frequently/often* vs. *sometimes/hardly* in function of whether the family had taken any trips to Poland since children’s third birthdays or not). No such link emerged, *χ*^2^(1, *N* = 28) = 0.020, *p* = 0.887.

The two questions about structural complexity within each language did not directly ask parents to compare across languages. Yet it was possible to compare the structural complexity ratings cross-linguistically for parents who had supplied ratings for each language (23 BFLA and 10 ESLA). As shown in Table 6, C, more than half the BFLA parents but only three ESLA parents indicated the same frequency of use of structural complexity across languages (e.g., the rating *often* for each language). Four BFLA and six ESLA parents gave higher structural complexity ratings for HL-Polish than SocL-German (e.g., *frequently* vs. *often*, or *often* vs. *sometimes*). Seven BFLA parents but only one ESLA parent gave higher ratings for SocL-German than HL-Polish (idem). Thus, the picture for ESLA differed from the one for BFLA, *χ*^2^(2, *N* = 33) = 6.130, *p* = 0.046: most ESLA parents rated children as more frequently producing complex HL-Polish than SocL-German structures, while most BFLA parents gave similar ratings in each language or rated children as more frequently producing complex SocL-German than HL-Polish structures.

The 23 BFLA parents who supplied structural complexity ratings for each language were parents to a total of 14 BFLA children. The 10 ESLA parents who supplied structural complexity ratings for each language were parents to a total of seven ESLA children. Focusing on the level of the children and abstracting away from double ratings for the same child^8^, it turns out that most of these ESLA children (five out of seven) but only three BFLA children received a higher complexity rating for HL-Polish (Table 6, D). On the other hand, nearly half (6/14) of the BFLA children had a higher complexity rating for SocL-German. This was the case for only a single ESLA child. There was one BFLA girl whose two parents agreed she often produced German complex structures but none in Polish (in separate communication, the mother commented that the girl had low HL-Polish speaking skills; she also had a bad score for HL-Polish comprehension). Five BFLA children but only a single ESLA child had the same complexity rating in both languages.

In summary, this section analyzed ratings of how often parents heard 3.5-year-olds produce the sort of complex structures expected for their age. Abstracting from a particular language, most children *frequently* or *regularly* produced complex structures and were thus developing language as expected. Maternal ratings for HL-Polish showed little variation amongst children. No BFLA-ESLA differences emerged here. Cross-linguistic comparisons both at the level of parental ratings and the level of children, however, did reveal a BFLA-ESLA difference: Most BFLA children’s use of complex utterances was rated similarly in both languages or higher in the SocL, whereas most ESLA children’s use of complex utterances was rated higher in the HL (with the caveat that comparisons at the child level were possible for only half the ESLA sample).

##### 3.1.2.2. Relative proficiency ratings: Parental comparative assessment of which language was better developed

The final proficiency questions asked parents explicitly (1) whether children spoke German better than Polish, and (2) whether children spoke Polish better than German (PR2 and PR5 in Appendix A). The question was asked in both directions in order to avoid skewed responses. Each two responses per parent were coded in terms of whether the responses were the same (indicating no difference between languages) or not. For differing responses that involved just *more or less agree* and *not quite agree* the language receiving *more or less agree* was coded as being better. Differing responses including at least one rating on the extreme were very clear as to which language was considered better and needed no additional coding. In no case were a parent’s answers for each language separately contradictory. All parents who had filled in BILTALK except one ESLA father answered both questions (this father only answered the question as to whether the child spoke better Polish than German). The two BFLA German-speaking fathers who had not answered the complexity questions (section 3.1.2.1) did answer these.

Table 7 lists the results according to individual responses by Polish-speakers and German-speakers as well as according to responses by a parent pair, where applicable. Two thirds of BFLA parents but none of the ESLA parents indicated there was no difference between languages. Ten BFLA parents identified SocL-German as the stronger language. Only one ESLA parent did. No BFLA parent claimed that their child spoke HL-Polish better than SocL-German. In contrast, all except one ESLA parent did. Differences between BFLA and ESLA for individual parental responses were statistically highly significant, *χ*^2^(2, *N* = 49) = 35.753, *p* < 0.001^9^.

**Table 7:** Which language did children speak best?

	According to…		
BFLA	Polish-speakers *N* = 14	German-speakers *N* = 11	Parent pair *N* = 11
HL-Polish	0	0	0
No difference	10	5	4
SocL-German	4	6	3
Parents disagreed	n.a.	n.a.	4
ESLA	Polish-speakers *N* = 22	German-speakers *N* = 0	Parent pair *N* = 9
HL-Polish	21	n.a.	9
No difference	0	n.a.	0
SocL-German	1	n.a.	0
Parents disagreed	n.a.	n.a.	0

n.a., not applicable.

Combining both BFLA parents’ ratings largely confirmed the individual response picture, although in four BFLA cases parents disagreed with each other, with Polish-speakers hearing no difference between languages, whereas German-speakers considered SocL-German better developed. All nine ESLA parent pairs agreed that HL-Polish was better developed.

Parental relative proficiency ratings showed a clear BFLA-ESLA difference. BFLA parents mainly indicated no difference between languages or higher proficiency in SocL-German. ESLA parents mainly indicated higher proficiency in HL-Polish. This result confirms tendencies earlier found for structural complexity. The following analysis combines structural complexity and relative proficiency ratings.

##### 3.1.2.3. Relative complexity and relative proficiency ratings combined

In a final analysis for proficiency comparative ratings for structural complexity and relative proficiency were combined (Table 8). This allowed for the tentative identification of different child proficiency profiles (tentative, because for 7 ESLA children there were no parental ratings for SocL complexity).

**Table 8:** Relative complexity and relative overall proficiency ratings combined (child level) *.

	BFLA	ESLA
**A.** Child proficiency profile: Similar performance in each language		
No cross-linguistic difference for both complexity and proficiency	5	0
**B.** Child proficiency profile: Tendency towards greater HL proficiency		
Higher HL complexity and proficiency	0	5
Higher HL complexity but cross-linguistically similar proficiency	1	0
No cross-linguistic difference for complexity but greater HL proficiency	0	2
Comparative complexity unknown but greater HL proficiency	0	6
Total	1	13
**C.** Child proficiency profile: Tendency towards greater SocL proficiency		
Higher SocL complexity and proficiency	2	0
Higher SocL complexity but cross-linguistically similar proficiency	3	0
No cross-linguistic difference for complexity but greater SocL proficiency	2	0
Comparative complexity unknown but greater SocL proficiency	0	1
Total	7	1
**D.** Child proficiency profile unclear		
Higher HL complexity but greater SocL proficiency	1	0

* Full ratings were available for all 14 BFLA children. Comparative complexity ratings were available for only 7 ESLA children because for the others no parent had supplied a German rating.

Child proficiency profiles showed a difference amongst BFLA and ESLA children. Five BFLA but no ESLA children showed similar performance in both languages. More BFLA than ESLA children tended towards greater SocL-German proficiency, and more ESLA than BFLA children tended towards greater HL-Polish proficiency, *χ*^2^(1, *N* = 22) = 14.20, *p* < 0.001. For one BFLA child it was impossible to decide on a proficiency profile because of contradictory parental ratings.

Combined findings for structural complexity and relative proficiency showed a clear difference between BFLA and ESLA children. BFLA children’s proficiency was generally the same across languages or better in SocL-German; ESLA children’s proficiency was better in HL-Polish.

#### 3.1.3. Comprehension and production compared

One may wonder whether there was any relation between comprehension (section 3.1.1) and production in terms of the language proficiency profiles in Table 8. Of the 10 children who occasionally misunderstood one or both their languages four showed less good comprehension in the language they spoke less well: Two BFLA children who occasionally misunderstood Polish spoke better German than Polish; likewise, but in the other sense, two ESLA children who occasionally misunderstood German spoke better Polish than German. This is what one might expect. Yet four ESLA children who occasionally misunderstood Polish spoke better Polish than German. One BFLA child who occasionally misunderstood Polish showed no difference between languages in production. An additional BFLA child who occasionally misunderstood either language spoke better German, and an ESLA child who did so spoke better Polish.

These variable results do not support any language balance link between comprehension and production.

### 3.2. Language choice patterns

This section examines language choice patterns amongst 3.5-year-olds and their parents. These patterns were queried through open ended overall questions in PEGEBOS-3 (listed in Appendix B) and detailed language choice questions in BILTALK (see Appendix D for the specific items and response categories). The same parents (25 BFLA and 23 ESLA, section 2.4) who filled in BILTALK items about child language proficiency responded to detailed language choice questions. A number of discrepancies arose amongst responses to overall and detailed questions. Before turning to those brief analyses are presented of language choice patterns that were only queried in PEGEBOS-3, *viz*., children’s language choice with siblings and self, language choice within the parent pair, and family language choice patterns outside the home. Overall language choice patterns had reportedly not changed since children’s third birthdays.

#### 3.2.1. Children’s language choice with siblings and self

Children’s language choice with siblings and self was queried through overall questions in PEGEBOS-3 (Appendix B). Both BFLA and ESLA children mostly spoke both languages with siblings (Table 9). If only a single language was used, it was HL-Polish. Likewise, in speech to self both BFLA and ESLA children used both languages. If only a single language was used, it was HL-Polish for ESLA children and SocL German for BFLA children.

**Table 9:** Children’s language choice with siblings and to self.

	BFLA	ESLA
**A**. Languages(s) target children speak with siblings *		
HL	2	6
Both HL and SocL	7	7
SocL	0	0
**B**. Languages(s) target children speak to themselves		
HL	0	3
Both HL and SocL	12	11
SocL	2	0

* 4 BFLA and 1 ESLA child were single children; 1 BFLA child had a younger baby sibling but was not speaking to the one-month-old yet. These children are not tallied here.

**Table 10:** Family language choice outside the home across five settings.

	BFLA	ESLA
Only or mostly HL-Polish	3	27
Mostly or only both languages	26	14
Only or mostly SocL-German	13	0

The basis for these numbers consists of frequency codes attributed to each speaker (28 children, 27 fathers, 28 mothers = 83 in total) across the five settings queried in Appendix B.

#### 3.2.2. Language choice within the parent pair

Parents’ language choice amongst each other was queried through overall questions in PEGEBOS-3 (Appendix B). BFLA families presented a variable picture in terms of language choice amongst mothers and fathers. Eight parent pairs spoke SocL-German with each other, one used HL-Polish. Four parent pairs used both languages (one of these also used English), and in the final family parents addressed each other in English. In contrast, not counting the ESLA single parent family, 20 ESLA parents in 10 families spoke only HL-Polish with each other. Three ESLA parents spoke both HL-Polish and SocL-German with their spouse (who in turn spoke just HL-Polish back; missing data for one parent).

#### 3.2.3. Overall language choice amongst family members in public

PEGEBOS-3 also asked to indicate which language(s) 3.5-year-olds and their parents spoke amongst themselves in five public settings (Appendix C). BFLA families were more likely to speak SocL-German outside the home than ESLA families, who mostly tended to use only HL-Polish (Table 10). Both family types did speak both languages outside the home as well, but ESLA families were far less likely than BFLA families to do so, *χ*^2^(2, *N* = 84) = 33.086, *p* < 0.001^10^.

#### 3.2.4. Parental language choice in interaction with target children

Table 11, A shows parental responses to overall parental language choice with their three-year-olds (based on PEGEBOS-3; Appendix A). A clear BFLA-ESLA difference emerged, with most BFLA children hearing the HL from one parent and the SocL from the other (pattern iv, see section 1.3), and most ESLA children hearing only the HL from both parents (pattern i, see section 1.3).

**Table 11:** Parental language choice with target children *.

	BFLA	ESLA
**A**. Parental language choice according to PEGEBOS-3		
(i) Both parents just the HL	0	13
(iii) Both parents both languages	0	1
(iv) One parent the HL, the other parent the SocL	13	0
(v) Both parents the SocL, one parent the HL	1	0
**B**. Parental language choice according to BILTALK: Detail		
Both parents just the HL	0	4
Both parents the HL and one parent the SocL	0	3
Both parents both languages	1	2
One parent the HL, the other parent the SocL	7	0
Both parents the SocL, one parent the HL	3	0
BILTALK data only for mother: Speaks the HL (same information as in PEGEBOS-3)	3	1
BILTALK data only for mother: Speaks both the HL and the SocL (information differs from the one in PEGEBOS-3)	0	3
BILTALK data only for mother: Speaks both the HL and the SocL (same information as in PEGEBOS-3)	0	1
**C**. Parental language choice according to BILTALK: Sole maternal BILTALK data absorbed		
(i) Both parents just the HL	0	5
(ii) Both parents the HL and one parent the SocL	0	7
(iii) Both parents both languages	1	2
(iv) One parent the HL, the other parent the SocL	10	0
(v) Both parents the SocL, one parent the HL	3	0

* Language choice pattern numbers refer to the ones outlined in section 1.3 earlier.

It was mothers who supplied overall parental language choice data, both for their own language choice and that of children’s fathers. Table 11, B shows results tallying maternal and paternal responses for parental language choice based on BILTALK (Appendix D). It also separately lists BILTALK data from eight mothers in the absence of paternal BILTALK data. Three of these mothers (all ESLA) earlier had stated they only spoke the HL to children but now indicated they spoke both the HL and SocL to them. Assuming that the language choice data they and the other five mothers had given for fathers was in fact correct, their data were absorbed in Table 11, C, yielding a picture that differs from the one in Table 11, A.

Seven ESLA children heard the HL from both parents and in addition the SocL from one parent (pattern ii, see section 1.3), a possibility that did not emerge according to overall language choice patterns. A clear BFLA-ESLA difference remained, though, with language choice patterns (iv) and (v) limited to BFLA families, and patterns (i) and (ii) limited to ESLA families. Instead of 13 “one parent, one language” BFLA families, however, there now appeared to be only 10. That shift was not quite as large, however, as the one for ESLA families, where instead of 13 families with both parents speaking only the HL to children (pattern i) there were in fact only five.

#### 3.2.5. Children’s language choice in interaction with their parents

Table 12, A shows parental responses to overall children’s language choice with their parents (based on PEGEBOS-3; Appendix B). A clear BFLA-ESLA difference emerged, with most BFLA children speaking the HL to one parent and the SocL to the other (pattern civ), and most ESLA children speaking only the HL to both parents (pattern ci).

**Table 12:** Children’s language choice with parents *.

	BFLA	ESLA
**A**. Child language choice according to PEGEBOS-3		
(ci) Only HL with both parents **	0	11
(cii) HL with one parent, both languages with the other	0	1
(ciii) Both languages with both parents	0	2
(civ) HL with one parent, SocL with the other	9	0
(cv) SocL with one parent, both languages with the other	5	0
**B**. Child language choice according to BILTALK: Detail		
(ci) Only HL with both parents **	0	4
(cii) HL with one parent, both languages with the other	0	4
(ciii) Both languages with both parents	3	2
(civ) HL with one parent, SocL with the other	5	0
(cv) SocL with one parent, both languages with the other	3	0
BILTALK data only from mother: Child speaks HL to her (same information as in PEGEBOS-3)	3	0
BILTALK data only from mother: Child speaks both languages to her (different information from PEGEBOS-3)	0	3
BILTALK data only from mother: Uninterpretable	0	1
**C**. Child language choice according to BILTALK: Sole maternal BILTALK data absorbed		
(ci) Only HL with both parents **	0	4
(cii) HL with one parent, both languages with the other	0	7
(ciii) Both languages with both parents	3	2
(civ) HL with one parent, SocL with the other	8	0
(cv) SocL with one parent, both languages with the other	3	0

* Child language choice patterns numbered to mirror parental language choice patterns numbers (Table 11).

** Or single parent, in one ESLA case.

Mothers had supplied overall children’s language choice data, both for children addressing them and their fathers. Table 12, B shows results tallying maternal and paternal responses for children’s language choice based on BILTALK (Appendix D). It also separately lists BILTALK data from seven mothers in the absence of paternal BILTALK data. Three of these mothers (all ESLA) earlier had stated children only spoke the HL to them but now indicated that children spoke both languages to them. Assuming that the language choice data they and three BFLA mothers had given for children’s language choice with fathers was in fact correct, their data were absorbed in Table 12, C (maternal BILTALK data for an additional ESLA mother were uninterpretable because of contradictory information), yielding a picture that differs from the one in Table 12, A.

Seven ESLA children spoke the HL to both parents and in addition the SocL to one parent (pattern cii), whereas according to overall language choice responses there was only one. A concomitant change was that far fewer (four instead of 11) ESLA children spoke just the HL to parents (ci). A clear BFLA-ESLA difference remained, with BFLA children showing two patterns (civ: HL to one parent, SocL to the other; cv: SocL with one parent, both languages with the other) that were not used by ESLA children, and ESLA children showing two patterns (ci: only HL; cii: HL with one parent, both languages with the other) that were not used by BFLA children. There were also three BFLA children who spoke both languages with both parents (pattern ciii), indicating increased use of the HL compared to the overall responses.

#### 3.2.6. Summary: Language choice findings

This section analyzed patterns of language choice from different perspectives, with a main focus on 3.5-year-olds. Children’s interactions with siblings and in speech to self mainly took place in both languages, regardless of whether children were growing up in a BFLA or ESLA setting. In speaking to their parents, 10 children used both languages with one parent, but only HL-Polish (seven ESLA children) or SocL German (three BFLA children) with the other. Five children spoke both languages to both parents. Twelve children exclusively used a single language with either of their parents. For eight BFLA children this single language was a different one for each parent. Considered from the perspective of individual parents, 23/54^11^ (0.43) were exclusively addressed in HL-Polish by their preschooler, 11/54 (0.20) in SocL-German, and 20/54 (0.37) were addressed in both languages. On the whole, then, interactional settings involving the HL (0.43 + 0.37) were more frequent than those involving the SocL (0.20). There were BFLA-ESLA differences here, however, with exclusive SocL usage with any parent limited to BFLA children. If ESLA children used the SocL with a parent, they were also using the HL with that same parent.

In many ways, children mirrored their parents’ language choice with them. Most parents (27/56, or a proportion of 0.48) addressed children in only the HL. Nearly a third (16/56; 0.29) used both languages with children (5 BFLA; 11 ESLA), and not even a quarter (13/56; 0.23) used only the SocL. Again, there were BFLA-ESLA differences here, with exclusive SocL usage to children by parents limited to BFLA families. With one exception families where both parents used the HL with children (regardless of whether they also spoke the SocL) were ESLA families.

The summary above for language choice amongst preschoolers and parents is based on responses to detailed language choice questions. These were often different from responses to overall language choice questions. As discussed further below, in case of internal inconsistencies amongst responses to overall versus detailed questions it is likely better to see the latter as more valid than the former. In addition, detailed language choice questions were often answered by two rather than just a single parent, thus increasing reliability as well.

Finally, family language choice outside the home was quite different for BFLA and ESLA families and reflected home language choice patterns amongst preschoolers and parents. The frequent use of two languages in the home was extended outside the home in BFLA families, whereas ESLA families tended to stick much more to just HL-Polish, in line with home language use. Use of just SocL-German outside the home only occurred in BFLA families.

## 4. Discussion and conclusion

This study sought to investigate whether young preschoolers with exposure to a heritage language (HL) from birth showed different patterns of HL development depending on whether they had in addition been exposed to another language from birth as well (RQ1). To this end the study compared HL development and use by two kinds of bilingual preschoolers: children who had heard two languages from birth in the home (Bilingual First Language Acquisition, BFLA) or children who had started off first hearing a single language at home and later added a second language through childcare or preschool (Early Second Language Acquisition, ESLA).

The HL in this study was Polish, and children were acquiring German as a societal language (SocL) either through home exposure from birth or through preschool. This specific focus on HL-Polish and SocL-German served to address RQ2. It fills a substantive gap in research on early HL-Polish development, not only in a German-speaking country, but also elsewhere: with the exception of Miękisz et al.’s (2017) study of toddlers, group studies on early HL-Polish development so far have focused on older preschoolers (e.g., Kulik, 2016; Haman et al., 2017; Mieszkowska et al., 2017; Abbot-Smith et al., 2018; Hansen et al., 2019).

Other than the BFLA-ESLA difference target children in this study were demographically comparable. Children were nearly all 3.5 years old. Gender distribution across BFLA-ESLA groups was similar. All children except one grew up in a dual parent family. All children except two grew up with at least one highly educated parent. If mothers worked outside the home, it was mainly part-time. Most fathers worked full-time outside the home. There were no BFLA-ESLA differences in parental ages. All Polish-speaking parents were born in Poland and had emigrated to the German-speaking country children lived in at the time of data collection. Most children were born in that same country. Being similarly aged, children had heard HL-Polish from at least one parent for the same length of time, i.e., from birth. They had the same experience in German-speaking preschool and were attending preschool by age three.

Data collected through a detailed online survey filled out by 28 mothers and 20 fathers revealed similarities but also important differences amongst BFLA and ESLA children (and their families).

Most children had no problems with comprehension in either language. In at least one language all except perhaps three children were saying complex utterances of the type generally expected for their age. No BFLA-ESLA differences emerged.

Most children regularly or frequently used complex HL-Polish utterances. This result is better than the one for 12 older Polish-German preschoolers (Kulik, 2016, p. 111). As Reich (2009) noted, four-year-olds may already start to stagnate in the HL. The present study did find one BFLA and two ESLA 3.5-year-olds who hardly used any HL-Polish complex structures. Whether HL stagnation was at work here will be examined in a future study comparing children’s performance on parent report data collected 9 months earlier. At any rate, contrary to findings by others (Slavkov, 2015), trips to the country where the HL is a SocL did not seem to have affected children’s HL proficiency.

When structural complexity ratings were compared across languages, BFLA-ESLA differences emerged, with BFLA children mostly not showing any difference between languages or doing better in SocL-German and ESLA children mostly doing better in HL-Polish. Additional parental ratings of which language they thought their child spoke better overall confirmed these differences. When these findings for relative overall proficiency were combined with those for structural complexity in one versus the other language the picture became even clearer: BFLA children’s proficiency was the same across languages or better in SocL-German; ESLA children’s proficiency was better in HL-Polish.

The finding of BFLA-ESLA relative proficiency differences already at age 3.5 shows the importance of taking into account that children’s exposure to the SocL from birth may lead to different HL trajectories than if exposure to the SocL started some time after birth. Studies of HL development in preschoolers that tally the age at which children first started being regularly exposed to the SocL often do not have a separate category for children who started such exposure at birth, thus potentially obscuring important differences amongst children: For instance, in their study of HL-Russian as used by 3.5- to 8-year-olds Gagarina and Klassert (2018) distinguished between children who were below 18 months, between 18 months and 3;05 years, or between 3;06 and 5;05 years old when they first came into regular contact with SocL-German; there was no separate category for children who started hearing SocL-German from birth.

These findings for preschoolers foreshadow differential findings for BFLA and (E)SLA primary school children with regard to HL use in the home: (E)SLA primary school children stand a far greater chance of speaking the HL than BFLA peers (see the Introduction). If BFLA preschoolers’ level in the SocL is better they are bound to want to use it more. The more they speak it, the higher their SocL proficiency will be. In contrast, bilingual children’s lesser use of a language may lead to declining proficiency in it (Ribot et al., 2018), and, ultimately, to children no longer speaking one of their languages (De Houwer, 2009).

Children’s use of a particular language forms part of their language choice patterns, that is, like all bilinguals, children always have to select one particular language when they speak, or use a mixed utterance combining material from both languages (De Houwer, 2019b). Recurrent language choice patterns with particular interlocutors lead to potentially highly unbalanced frequencies of use of a particular language. Thus it is important to gain reliable information about language choice patterns, not only those of children, but also those of parents, who, aside from staff and peers in group settings such as preschool, are children’s main providers of language input from and through which children acquire their languages.

This study used different ways of querying language choice patterns. Some discrepancies between general and detailed questions about language choice were to be expected (section 1.5). Furthermore, the addition of information sources by having data supplied by both parents for 20 of the 28 families was also expected to lead to discrepancies with information provided by a single person. However, the magnitude of the discrepancies between answers by a single parent (mothers) about their and family members’ overall language choice and parental answers by each parent on detailed questions regarding their own language choice and that of their children in interaction with them was not expected. Especially for ESLA families discrepancies were surprisingly large. Compared to overall information, many more ESLA parents and children spoke both languages with each other rather than solely HL-Polish. Parental questionnaires about bilingual children do not usually highlight the respondents (e.g., the in-depth review by Kašćelan et al., 2022, does not mention anything about who filled out questionnaires except that “the information tends to be obtained from parents/caregivers, teachers, and to a lesser extent from the children themselves”, p. 29), and do not normally contain items querying the same information in different ways. Researchers should be aware that general information supplied by a single parent may not reflect actual practice. One can assume that answers to more detailed questions by more than a single respondent, as done in this study by involving both members of a parent pair, give a more accurate and reliable picture.

In the present study, BFLA mothers mostly espoused a “one parent, one language” setting as far as overall parental language choice with preschoolers was concerned, and indicated that children followed a parent’s language choice or spoke SocL-German with one parent and both languages with the other. This picture was more or less confirmed by the detailed information, although that information showed a few more parents and children using both languages with each other rather than just a single one. In contrast, ESLA mothers mostly presented their family as speaking exclusively HL-Polish but in as many as half of ESLA families detailed information showed parents speaking both languages to children. In only half of ESLA families was the information for children’s language choice the same across overall and detailed information. The larger discrepancies for ESLA families on the one hand and the smaller discrepancies for BFLA families on the other can perhaps be explained by different life circumstances of mothers in each type of family. Mothers in BFLA families have been part of a bilingual family since their child was born and were perhaps more aware of linguistic choices from the very start and much more focused on language use than mothers in ESLA families. Hence they were able to give general language choice information that was much closer to actual practices. One could also imagine that being part of a fully Polish origin family rather than a transnational family in the case of BFLA mothers supported ESLA mothers’ sense that only HL-Polish was part of family life, and that it was only when they were asked to reflect on detailed practices that they considered actual reality, which included much more home SocL use than was reported in a general fashion (in their study of HL-Polish-speaking families with ESLA toddlers in the UK using detailed language choice questions Miękisz et al., 2017 found that SocL-English use at home was not uncommon).

Children’s language choice patterns were quite distinct for BFLA and ESLA families. BFLA children used HL-Polish in fewer interactional settings with parents than did ESLA children. ESLA children spoke HL-Polish with both parents. Those BFLA children who did so also spoke SocL-German with both parents and thus divided up the time between languages. Several ESLA children spoke both languages with one parent as well but still spoke HL-Polish only with the other parent. Most BFLA, but not ESLA, children exclusively spoke SocL-German with at least one parent. Although these language choice patterns do not say anything with regard to actual frequency of use, they are quite different, and are a direct result of children growing up within very different home language environments. ESLA children’s more varied use of HL-Polish in the family compared to BFLA children may help explain the fact that they were more proficient in HL-Polish than SocL-German compared to BFLA children.

The fact that BFLA and ESLA children’s home language environments were in fact quite distinct is clear from the detailed parental language choice data for their interactions with preschoolers. No ESLA parent spoke only SocL-German with children, but many BFLA parents did. Double as many ESLA than BFLA parents spoke both languages with children: More so than ESLA parents, BFLA parents stuck to a single language with children. The fact that all ESLA children heard HL-Polish from both parents and most BFLA children only from a single parent suggests that exposure to HL-Polish was generally more varied in the ESLA families and could help explain ESLA children’s higher HL-Polish proficiency, in addition to the fact that ESLA children themselves spoke HL-Polish in more interactional settings (*cf*. above). Some authors assume that children growing up with just the HL at home hear more of it at home than families growing up with both the HL and the SocL at home (*cf*. Rodina and Westergaard, 2017; Rodina et al., 2020; see also Flores et al., 2017, who assume that “children who are growing up in Portuguese–German households have significantly less exposure to their HL than children whose HL is the dominant language spoken at home, even though both groups are exposed to Portuguese from birth,” p. 809). However, findings about parental language choice do not say anything about the absolute frequency of parental input in each language, nor about the proportion of use of each language. It is an empirical question as to whether there is a default difference in the frequency of home HL exposure in bilingual rather than monolingual HL-speaking families. Children growing up in BFLA versus ESLA families do have a very different language ecological experience with each language (De Houwer, 2018b, 2021). For instance, for BFLA but not ESLA children large linguistic variation in the input is present from the outset, and BFLA but not ESLA children have been used to people speaking in fundamentally different ways from birth. BFLA but not ESLA children have learned to understand words and expressions in both languages from early infancy onwards, and BFLA but not ESLA children’s early language production usually is distributed over two languages. BFLA families have emotional and cultural connections with two languages and personal connections with speakers of each; this is quite different for ESLA families, whose connections are mainly tied to a single language. All this helps explain why by age 3.5 this study already found differences between BFLA and ESLA children’s development and use of the heritage language.

A question not usually asked in studies of young bilinguals is which language(s) children speak to themselves (see Sawyer, 2016, for a review of the few studies investigating bilingual children’s private speech). In the current study bilingual preschoolers overwhelmingly used both their languages in private speech. This finding merits further exploration. For instance, it would be interesting to know whether different private speech functions are associated with a different language, or whether the self-regulatory functions associated with private speech are used regardless of language.

In addition to the general question about children’s language choice with themselves there were general questions about children’s language choice with siblings, language choice amongst parents, and family language use outside the home. Answers to these questions are presumably less likely to have personal feelings of identity and investment attached to them than questions regarding parental language choice with children and children’s language choice with parents. Therefore they are likely quite reliable. Especially the question about language use outside the home was quite complex and required nuanced and focused answers, much like the detailed language choice questions. Also, it is unlikely that parents have fixed ideas about what language(s) they should be using outside the home. It was striking that the BFLA-ESLA input difference as obtained through detailed language choice questions was reflected in family language practices outside the home, thus reinforcing BFLA children’s greater use of and exposure to SocL-German, and ESLA children’s greater use of and exposure to HL-Polish.

A final point relates to the method used to gain information about children’s HL proficiency. Resources did not allow the collection of observational data as would be possible through the use of the MAIN approach, for instance (MAIN: Multilingual Assessment Instrument for Narratives, first described in Gagarina et al., 2012), which has been standardized both for Polish and German (in addition to many other languages). Standardized parental questionnaires to help assess language proficiency exist, but are only usable with children until the end of the third year (Polish: Smoczyńska, 1999/2015; German: Szagun et al., 2009). In the same spirit as those parental questionnaires it was decided to include six survey questions to gain an idea of children’s language proficiency. The fact that the answers yielded fairly consistent results can be seen as a validation of the basic method. Abbot-Smith et al. (2018) showed for 20 even older Polish-English bilinguals (five-year-olds) that parental reports on child language proficiency matched results from laboratory-based tests, and thus were reliable sources of information. However, for better reliability it would be best to include additional questions.

The reliability of the parental reports in the present study was likely enhanced by including both maternal and paternal ratings of child proficiency, a fairly unique method compared to most studies of bilingual preschoolers (but see Lundén and Silvén, 2011; Byers-Heinlein and Werker, 2013; De Houwer et al., 2014). This gave a fuller picture of children’s language development than could have been obtained solely on the basis of maternal report. Some of the results confirmed that in order to properly evaluate whether bilingual children are overall developing language as expected it is useful to have both parent reports (see also De Houwer, 2019a): As regards structural complexity, a less favorable report by one parent for one of the languages was sometimes offset by a more favorable report by the other parent for the other language. This was true both for BFLA and ESLA children. Although there were some minor differences amongst parental ratings in the same family, parents mostly agreed with each other, in spite of sometimes large differences between mothers’ and fathers’ levels of Polish and German proficiency in the BFLA and ESLA families, respectively.

In spite of the rich data gathered through involving both mothers and fathers in data collection, both the relatively small number of children reported on in each group and the large similarity amongst children on several measures resulted in only suggestive results regarding links between children’s relative language proficiency levels on the one hand, and child language choice on the other. Also, the current analyses focused on several factors by themselves. It is likely that clusters of factors may need to coagulate in order for strong links to emerge. For instance, three-year-olds who speak both languages to HL-speaking parents AND attend SocL preschool for more than the average number of hours AND have not recently been to the country where the HL is spoken may stand a much greater chance of performing worse in the HL. Studies with larger groups of children are needed to investigate this. Furthermore, absolute and relative amounts of input in both languages, not investigated in the present study, are important additional factors to take into account (De Houwer, 2018c).

Most parents reported that language choice patterns with children had not changed since children’s third birthdays, mirroring similar findings in De Houwer and Bornstein (2016). The Polish-speaking parents of BFLA preschoolers with better German than Polish skills who also spoke German with their Polish-speaking parents may find that with time, it becomes difficult to continue to speak Polish to children. They may eventually switch to German, thus effectively stopping the support for Polish. How Polish-speaking parents respond to preschoolers’ use of German may thus be crucial in helping to determine whether children will continue to speak Polish in Germany. As Pułaczewska (2018, 2019) has documented, many Polish-German adolescents do not. More knowledge of factors explaining young bilinguals’ variable language proficiency profiles will help Polish-German families in particular and bilingual families in general to support the continued development of the heritage language.

## Data availability statement

The raw data supporting the conclusions of this article will be made available by the authors, without undue reservation.

## Ethics statement

Ethical review and approval was not required for the study on human participants in accordance with the local legislation and institutional requirements. The patients/participants provided their written informed consent to participate in this study.

## Author contributions

AD designed the study and closely oversaw data collection and initial data coding (see Acknowledgements), carried out all specific coding for this study and its analyses, and wrote the manuscript.

## Funding

The University of Erfurt and the Harmonious Bilingualism Network provided the funding.

## Conflict of interest

The author declares that the research was conducted in the absence of any commercial or financial relationships that could be construed as a potential conflict of interest.

## Publisher’s note

All claims expressed in this article are solely those of the authors and do not necessarily represent those of their affiliated organizations, or those of the publisher, the editors and the reviewers. Any product that may be evaluated in this article, or claim that may be made by its manufacturer, is not guaranteed or endorsed by the publisher.

## Footnotes

^1^The author prefers the term "non-societal language" but uses the term "heritage language" in the framework of the present Special Issue.

^2^These categories were made on the basis of survey data specifying which language(s) each parent in a family spoke at home on the whole. There were no data on what language(s) parents specifically addressed to children or each other.

^3^With the caveat that some parents in monolingual HL-speaking families may have started to also speak the SocL at home after children started attending school.

^4^Information on the ages at which the children in De Houwer (2007) who did not hear the SocL at home first started attending (pre)school in the SocL is unavailable. Given the fact that nearly all children in Flanders attend preschool most children who did not hear the SocL at home likely started hearing the SocL in preschool, and were thus ESLA children.

^5^A notable exception is Czapka et al. (2021), who included children aged 2;1 to 4;1 at pretest (mean of 3;3). Note, however, that the range here is quite large, with some children having lived twice as long as others at pretest, thus potentially masking developmental differences within this wide age range.

^6^The terms "Polish" and "German" are meant to refer to the respective languages unless otherwise noted.

^7^In Germany one speaks of "Kindertagesstätten" (KiTas) when such a preschool offers all day care and education, and of "Kindergärten" when children stay for only half a day. Neither of these forms is part of the formal school system, which is only available for children aged six and up.

^8^For the 20 children whose two parents supplied a structural complexity rating the best rating was listed in Table 6, D (e.g., if father gave a higher frequency rating for one language compared to the other, and mother gave similar ratings, regardless of the level, father’s higher frequency rating was counted). For seven children there was no difference amongst maternal and paternal ratings; for six children fathers gave higher ratings, and for seven children mothers gave higher frequency ratings.

^9^Because two cells in the contingency table equaled zero their values had to be raised to 1 for statistical treatment. The actual *N* was 47.

^10^Because one cell in Table 10 equaled zero its value had to be raised to 1 for statistical treatment.

^11^The 28 children had 56 parents but in one case there was no contact with the father and in another case child language choice data were contradictory.

## Supplementary material

Supplementary Data Sheet 1 - Appendices A-D

Appendix A: Language proficiency questions.

Appendix B: Overall language choice questions in PEGEBOS-3.

Appendix C: Overall language choice questions in PEGEBOS-3 about language choice in public.

Appendix D: Detailed language choice questions.

## Abbreviations

BFLA: Bilingual First Language Acquisition
BILTALK: sub-questionnaire of the PEGEBOS-3 survey filled out by both parents
ESLA: Early Second Language Acquisition
HL: heritage language
PEGEBOS-3: online survey used for data collection
SocL: societal language
