# Supplementary material for: Polish-German preschoolers develop and use heritage Polish differently depending on whether they heard German from birth or not
Source: Front Psychol. 2023 Mar 28;14:1080122. doi: 10.3389/fpsyg.2023.1080122 (PMC10086410; doi:10.3389/fpsyg.2023.1080122)
Supplement: Supplementary Data Sheet 1 - Appendices A-D [file Data_Sheet_1.pdf]

Appendix A. Language proficiency questions

(excerpted from BILTALK, English translation)

All items here appeared in the order below but were interspersed with items in Appendix D and additional items not covered in the current study. Parents responded to statements about each language. Similar proficiency questions did not immediately follow each other. Items are numbered here for easy reference but were not numbered in the parent survey.

IX. Talking with your child

This is the part where we need both mother and father!

We would like to know more about how you and your child talk to each other. Some of the statements might appear repetitive to you, but please bear with us, and answer all of them! Thank you.

Mother talking with child (The "I/me" here is mother) [separate but identical set of questions also asked of the father, not listed here]

(PR1) When I talk to my child in German, (s)he often expresses misunderstanding of a word or phrase

Completely agree
More or less agree
Not quite agree
Entirely disagree
Doesn't apply, I don't speak any German to my child]

(PR2) As far as I can be a judge of it, my child speaks much better Polish than German

Completely agree
More or less agree
Not quite agree
Entirely disagree

(PR3) When I talk to my child in Polish, (s)he often expresses misunderstanding of a word or phrase

Completely agree
More or less agree
Not quite agree
Entirely disagree

Doesn't apply, I don't speak any Polish to my child]

(PR4) I've heard my child say Polish sentences like: "a jak wiewiórka umrze, to gdzie idzie?" or " ja najpierw muszę przyjść do pani " or "daj mi te pieniążki, bo ja będę zapłacać" (please don't pay attention to the actual words, but to the overall structure)

Hardly

Very often, and more complicated ones, too

No

Once in a while

Regularly

(PR5) As far as I can be a judge of it, my child speaks much better German than Polish

Completely agree

More or less agree

Not quite agree

Entirely disagree

(PR6) I've heard my child say German sentences like: "erst muss ich mich mal richtig hinlegen" or "komm, wir wollen dies gerade spielen" or "Mutti, ich hätte im Spiel Fieber, weil meine Stirne sind ganz heiss" (please don't pay attention to the actual words, but to the overall structure)

Hardly

Very often, and more complicated ones, too

No

Once in a while

Regularly

Appendix B. Overall language choice questions in PEGEBOS-3

(excluded here: questions about LC with children's grandparents; English translation)

Current patterns of language use amongst family members

Please fill in which language a particular family member speaks to another one when there are only family members present. You may fill in more than a single language. Please also indicate what language(s) your child speaks to herself/himself.

Please write in N/A if a particular person is not part of your household/family (e.g., there is no twin). Family members are specified from your child's perspective.

Your child

Speaks to father _______________

Speaks to mother _______________

Speaks to self _______________

Speaks to older sister(s) _______________

Speaks to baby sister(s) _______________

Speaks to older brother(s) _______________

Speaks to baby brother(s) _______________

Mother

Speaks to father _______________

Speaks to your child _______________

Speaks to older sister(s) _______________

Speaks to baby sister(s) _______________

Speaks to older brother(s) _______________

Speaks to baby brother(s) _______________

Father

Speaks to mother _______________

Speaks to your child _______________

Speaks to older sister(s) _______________

Speaks to baby sister(s) _______________

Speaks to older brother(s) _______________

Speaks to baby brother(s) _______________

Have there been any big changes in language use amongst family members since your child´s third birthday?

Yes/No

[skip logic possibilities in the survey platform jumped to the next question if respondents answered "no":]

Please explain:

____

Appendix C. Overall language choice questions in PEGEBOS-3 about language choice in public

(English translation)

Current patterns of language use outside the home

Please fill in which language mother, father and your child speak amongst each other **outside** the home, when others are present as well. You may fill in more than one single language.

*The language **your child** uses with mother and/or father*

In a restaurant _______________

While in a park or playground _______________

In the company of friends _______________

At a party with extended family _______________

When shopping in a supermarket or mall _______________

*The language **mother** uses with the child and/or father*

In a restaurant _______________

While in a park or playground _______________

In the company of friends _______________

At a party with extended family _______________

When shopping in a supermarket or mall _______________

*The language **father** uses with the child and/or mother*

In a restaurant _______________

While in a park or playground _______________

In the company of friends _______________

At a party with extended family _______________

When shopping in a supermarket or mall _______________

Appendix D. Detailed language choice questions

(excerpted from BILTALK, English translation)

All items here appeared in the order below but were interspersed with items in Appendix A and additional items not covered in the current study. Parents responded to statements about each language. Similar LC statements did not immediately follow each other.

All items here had as response categories:

Completely agree

More or less agree

Not quite agree

Entirely disagree

The weaker agreement there was with a statement the less applicable it was.

IX. Talking with your child

This is the part where we need both mother and father!

We would like to know more about how you and your child talk to each other. Some of the statements might appear repetitive to you, but please bear with us, and answer all of them! Thank you.

Mother talking with child (The "I/me" here is mother) [separate but identical set of questions also asked of the father, not listed here]

I always speak just German to my child

My child always speaks just German with me

I always speak just Polish to my child

My child always speaks just Polish with me
